# Antiretroviral Activity Of a Novel Pyrimidyl-Di(Diazaspiroalkane) Derivative

**Published:** 2017

**Authors:** E.A. Novoselova, O.B. Riabova, I.A. Leneva, V.G. Nesterenko, R.N. Bolgarin, V.A. Makarov

**Affiliations:** «NEARMEDIC PLUS» LLC, Aviakonstruktora Mikoyana str. 12, Moscow, 125252, Russia; A.N. Bach Institute of Biochemestry, Federal Research Centre «Fundamentals of Biotechnology» of the Russian Academy of Sciences, Leninskiy pr. 33-2b, Moscow, 119071, Russia; I.Mechnikov Research Institute of Vaccines and Sera, Malyj Kazennyj per. 5, Moscow, 105064, Russia

**Keywords:** antiviral activity, dispiro compounds, heparan sulfate, HIV

## Abstract

A novel compound, 3,3’-(5-nitropyrimidine-4,6-diyl)bis-3,12-diaza-6,9-diazoniadispiro[5.2.5.2]hexadecane tetrachloride dihydrochloride, was synthesized. The compound was found to inhibit the replication of various viral families by blocking specific heparan sulfate receptors on the host cell’s surface. In experiments, the compound was found to be highly effective against several strains of HIV retroviruses.

## INTRODUCTION

Searching for compounds that could block the interaction between a pathogen and the host cell is one of the promising directions in designing both antiviral and antimicrobial drugs. This approach has several advantages, especially in the case of antiviral drugs, since there is no need for the active compound to penetrate into the cells, which dramatically reduces both the cytotoxicity and overall toxicity of the substance used.

At the first stage of viral infection development, the virus adheres to the cell through the binding of specific viral proteins to specific molecules on the cell surface. Most often, adhesion occurs through specific binding to heparan sulfate proteoglycans (HSPG), which are located on the cell surface. It is known that this mechanism is used by various viral families, such as type 1 and 2 herpesviruses (HSV-1, HSV-2) [1], papillomaviruses (HPV) [2], human cytomegalovirus (HCMV) [3], some strains of the human immunodeficiency virus (HIV) [4], human respiratory syncytial virus (HRSV) [5], the hepatitis B and C viruses (HBV and HCV) [6], and others.

Previously we synthesized N,N-bis(1-oxido [1,2,5] oxadiazolo [3,4] pyrimidin-7-yl)-3,12-diaza-6,9-diazonium (5,2,5,2) dispirohexadecane dichloride **1 **(*Figure*), the most well known and most extensively studied inhibitor of the adhesion process. It was shown that compound **1 **and its analogues, including dispirotripiperazine, are characterized by effective reversible binding to cellular HSPG and, thus, prevent virus binding [7]. Dispiro compound **1 **inhibits replication in herpes viruses [8], as well as other viral families that use HS as a receptor or co-receptor [3]. However, the metabolic instability of compound **1 **that is due to a degradation accompanied by the formation of nitric oxide prohibited an investigation of its biological properties [9].

Therefore, we attempted to obtain a novel dispirotripiperazinium derivative. We designed an optimal compound capable of binding to known HSPG with allowance for a potential metabolic stability of the target compound and synthesized 3,3’-(2-methyl-5-nitropyrimidine- 4,6-diyl)3,12-bis-6,9-diaza-diazoniadispiro [5.2.5.2] hexadecane tetrachloride dihydrochloride 2. It was assumed that dispiropiperazine 2, which is represented in a more chemically stable structure, would be a similarly effective blocker of cellular HSPG and, thereby, would inhibit cell adhesion of the virus, leading to a disruption of the life cycle and reduced titer of the virus.

**Fig. 1:**
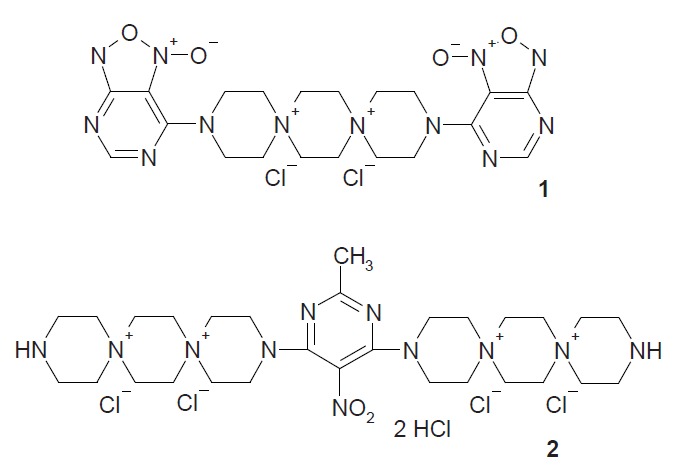
The structure of N,N’-(bis-1-oxido[1,2,5]oxadiazolo [3,4d]-nitropyrimidine-7-il)-3,12-diaza-6,9-diazonia( 5,2,5,2)dispirohexane dichloride (1) and 3,3’-(5-nitropyrimidine-4,6-diyl)bis-3,12-diaza-6,9-diazoniadispiro[ 5.2.5.2]hexadecane tetrachloride dihydrochloride (2).

In this paper, we report on the high antiviral activity of the novel dispirotripiperazinium derivative **2, **which fully confirms our hypothesis. Currently, dispiro compound **2 **is undergoing in-depth preclinical studies.

## EXPERIMENTAL

Our studies were carried out at the National Cancer Institute (National Cancer Institute, Bethesda, Maryland, USA). Antiviral activity was assessed using the XTT test [10] with various concentrations of the compound and incubation of cells (CEM-SS, MT-2, MT-4) in the presence of serial dilutions of dispiropiperazine 2 (dissolved in dimethyl sulphoxide, DMSO) for 6 days at a temperature of 37°C and humidified atmosphere with 5% CO_2_, followed by addition of XTT reagents. The method is based on a spectrometric measurement of the level of formazan transformed by living cells from a water-soluble tetrazolium salt, XTT.

**Table 1:** Antiviral activity of dispiro piperazine 2 against different viral strains

Viral strain	Cell line	CC50,μM	IC_50_,μM	TI (CC50/IC_50_)
HIV-1 6S	MT-2	98.2	1.4	71.4
HIV-1 IIIB	MT-4	200.0	5.7	35.0
HIV-1 RF	CEM-SS	197.0	150.0	1.3
HIV-1 N119	MT-4	200.0	31.7	6.3
HIV-1 DPS	MT-4	200.0	1.2	170.0
HIV-1 A-17	MT-2	79.1	4.7	16.7
HIV-1 A-17	MT-4	200.0	33.7	5.9
HIV-2 ROD	CEM-SS	200.0	13.3	15.0
SIV MAC 251	MT-4	200.0	6.3	31.5

Note: CC_50_ – cytotoxic concentration; IC_50_ – half-maximal inhibition concentration; TI – therapeutic index.

The following HIV strains were used: the reference HIV-1 IIIB strain; the AZT-sensitive HIV-1 6S, HIV- 1 RF strains; the drug-resistant HIV-1 N119 strains (resistant to nevirapine, Y181C mutation), HIV-1 DPS (resistant to diphenyl sulfone, Y181C mutation), and HIV-1 A-17 (resistant to nevirapine, K103N and Y181C mutations). We also used the HIV-2 ROD strain and simian immunodeficiency virus SIV MAC 251.

## RESULTS AND DISCUSSION

The results, summarized in *Table, *show that dispirotripiperazine **2, **which we synthesized, effectively inhibits the replication of HIV-1, HIV-2, and SIV. At the same time, in contrast to the previously obtained data on the activity of compound **1 **against herpes viruses, we observed a fairly wide range of sensitivity of HIV and SIV.

HIV-1 and HIV-6S 1 DPS were the most sensitive to the inhibitory effects of dispiro compound **2 **among the tested strains, IC_50_ = 1.37 and 1.17 μM, respectively. In contrast to these strains, HIV-1 RF was 100-fold less sensitive to the test compound (IC_50_ = 150 μM).

Unlike HSV viruses, wherein the IC_50_ values were within the same range, as well as the values for all tested strains (8.2 to 20.4 μM), inhibition of HIV replication heavily depended on the used strains, where the IC_50_ values varied from 150 μM (HIV-1 RF, CEM-SS cell line) to 1.4 μM (HIV-1 6S, MT-2 cells). In the case of the HIV-1 A-17 strain, IC_50_ values were determined for different cell lines and they ranged from 33.7 μM for the MT-4 line to 4.7 μM for the MT-2 line.

Such a significant difference in IC_50_ values (both within a single cell line for different HIV strains, and within a single strain for different cell cultures) may be due to two reasons. First, different cell lines may have a different surface concentration of heparan sulfate proteoglycans. Second, it has been reliably shown that HIV strains differ significantly from each other in the efficiency of co-receptor use (CCR5 and CXCR4) at the stage of target cell binding [11].

Unlike the herpes viruses, the structure of HIV binding mediated by heparan sulfate has not yet been confirmed by an X-ray analysis, and the possibility of interaction between HIV and the host cell, mediated by heparan sulfate proteoglycans, has only been reported in the literature.

We believe that the new class of pyrimidyl-di(diazodispiroalkane) derivatives can be used as antiviral agents. Our belief is rooted in several factors, such as the specificity of the inhibitory effect with respect to viral strains, as exemplified by dispiro tripiperazinium **2**; the ability of dispiro compounds to form very stable bonds with some viral receptors or co-receptors; and the composition with a chemically defined low molecular weight [7].

It should be noted that this mechanism of action is very promising in terms of overcoming the resistance shown by viruses toward officinal medicines, since it acts on the cell rather than on the virus itself. Owing to these properties, this class of compounds can be a valuable tool in studying virus-cell interactions.
